# A Case Report of Klippel-Feil Syndrome Presenting as Tetraplegia

**DOI:** 10.7759/cureus.41241

**Published:** 2023-06-30

**Authors:** Madineni Bhavana Chowdary, Manohar S, Dinesh Kumar, Athish KK

**Affiliations:** 1 General Surgery, RL Jalappa Hospital and Research Centre, Kolar, IND; 2 Neurosurgery, Sri Devaraj Urs Medical College, Kolar, IND; 3 General Surgery, Sri Devaraj Urs Medical College, Sri Devaraj Urs Academy of Higher Education and Research, Kolar, IND; 4 Internal Medicine, Sri Devaraj Urs Academy of Higher Education and Research, Kolar, IND

**Keywords:** screening, spinal trauma, cervical vertebrae, ­trauma, paraplegia

## Abstract

Cervical spine assessment is an important step in patients who sustained trauma. Klippel-Feil syndrome (KFS) is a complex syndrome with an abnormal fusion of cervical vertebrae at C2 and C3, which is caused by a failure in the division or normal segmentation of the cervical spine vertebrae in early fetal development. This condition leads to a characteristic appearance of a short neck, low hairline, facial asymmetry, and limited neck mobility. People with congenital defects like KFS are more prone to cervical spine injury. KFS is a relatively rare disease. Trivial trauma can lead to neurologic symptoms in such individuals. We present a 32-year-old male, with an alleged history of falls from height with traumatic injury to his head and spine. Following the event, he was unable to move all four limbs. The patient’s short neck was noted. Magnetic resonance imaging (MRI) of the spine revealed multilevel fusion of four cervical vertebrae (C3-C7) with a single fused spine which is highly uncommon. Myelopathy secondary to C3-C4 disk protrusion is also seen. The patient was diagnosed with KFS and managed conservatively. The abnormal fusion in KFS predisposes to neurologic injury owing to altered biomechanics of the spine leading to hypermobility of the adjacent normal spine, spondylolisthesis, and stenosis, thereby increasing the likelihood of injuries. Screening and identification of KFS in young children are essential as counseling for lesser strenuous activity might avoid neurological injury and promote better neurological outcomes in the future.

## Introduction

Cervical spine assessment is an important step in patients who sustained trauma. The basic dictum in the emergency room (ER) is that all patients who have sustained physical trauma are considered to have cervical spine injuries until proven otherwise. People with congenital defects like Klippel-Feil syndrome (KFS) are more prone to cervical spine injury [1].

KFS was first described in 1912 and was defined as a congenital anomaly with a triad of a short neck, low hairline, and limited neck mobility. Although less than 50% of the patients have all the classical triad [1], it is difficult to estimate the incidence and prevalence of KFS as it goes undiagnosed in many patients due to the asymptomatic state [1,2].

Even a trivial trauma can lead to neurologic symptoms in individuals suffering from KFS, and in a few cases, this may even lead to the diagnosis of KFS in previously undiagnosed individuals [3]. We report a case of quadriparesis in a young male following minor trauma who was later diagnosed with KFS.

## Case presentation

A 32-year-old gentleman was brought to the ER, with an alleged history of a fall from a height of 20 ft while he was performing a domestic chore on his terrace. After the incident, he was unable to move all four of his limbs. There was no history (post-event) of antegrade/retrograde amnesia, vomiting, loss of consciousness, dizziness, or any bleeding from the ear/nose/throat. There was no significant past history of trauma.

On examination, the patient was found to be conscious, oriented to time, place, and person, and receptive. Vitals were stable with pulse recording of 87/min, blood pressure of 120/70 mmHg, respiratory rate of 20 breaths/min, a saturation of 95%, GCS (Glasgow coma scale) of 9/15 E4 V5 M0, and Karnofsky performance status of 30%. General physical examination showed no pallor, no cyanosis of limbs, and no peripheral skin lesions, with all peripheral pulsations equally felt.

A neurological examination of the higher mental function and cranial nerves was within normal limits. The patient was assessed and graded as modified Nurick’s grade 6 and AIS B injury (American Spinal Injury Association Impairment Scale). Sensory deficits below the level of nipple and quadriplegia were noted. He had a grade "0" on the manual muscle testing (MMT) method, with a normal tone and areflexia.

Based on the current clinical findings, the patient was diagnosed to have a provisional diagnosis of traumatic cervical myelopathy. Post-emergency triaging, neck immobilization was done with a Philadelphia collar; the bladder was catheterized and was started on intravenous methylprednisolone as a neuroprotective agent. During the secondary survey, the patient was noticed to have a short neck for which the patient had no prior history.

**Figure 1:**
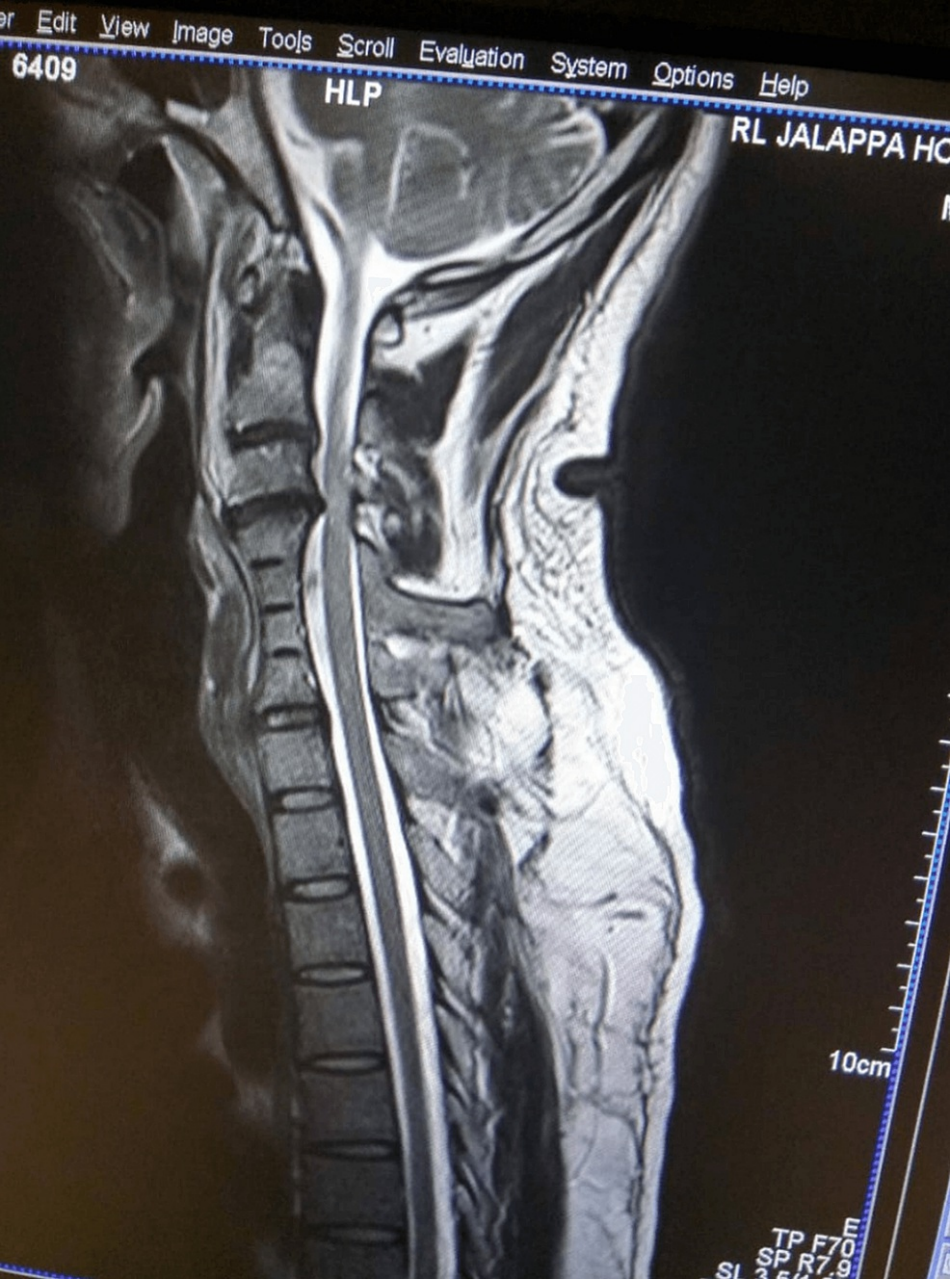
Sagittal section showing the block vertebrae, compressive myelopathy secondary to C3-C4 disk protrusion C: Cervical spine.

**Figure 2:**
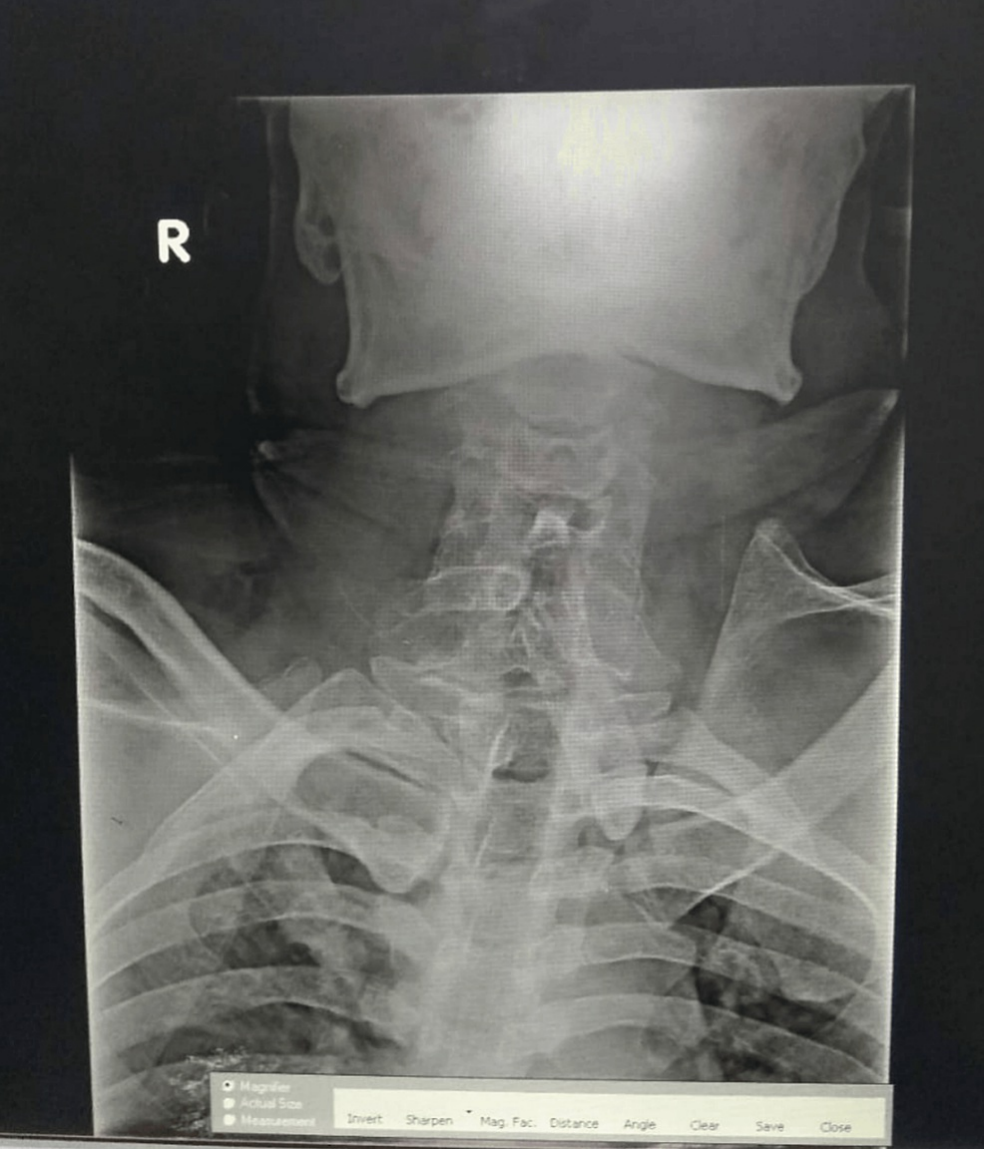
X-ray of the neck showing the fused cervical vertebra and large spinous process

The patient was stabilized and subjected to radiological investigation of an x-ray chest and neck. Based on the findings on the neck x-ray, it was followed by an MRI (magnetic resonance imaging) of the spine (Figure 1), which revealed the following block vertebrae, compressive myelopathy secondary to C3-C4 (cervical spine) disk protrusion, and x-ray showing fused cervical vertebrae and large spinal process (Figure 2). The patient was managed conservatively with neck immobilization, intensive care for a day followed by supportive management for two weeks. He recovered gradually with modified Nurick’s grade 4 and AIS D and was discharged on home advice and physiotherapy. He was followed up for six months and a year. There was a good recovery with improvement to MMT grade "3" at six months, and he was able to perform sedentary activities for almost one year. Family members were requested for a genetic analysis for future prospects, but compliance was not good due to financial constraints.

## Discussion

KFS is a relatively rare disease characterized by a triad of short neck, low posterior hairline, and limited neck mobility due to abnormal fusion of adjacent cervical vertebrae, with all three present in less than 50% of these patients [1]. The rest of the patients without all three signs are considered to have the Klippel-Feil variant [1]. The abnormal fusion of vertebrae may include the fusion of cervical spines. This abnormal fusion predisposes those with KFS to neurologic injury owing to altered biomechanics of the spine, leading to hypermobility of adjacent normal spine, spondylolisthesis, and stenosis and thereby increased likelihood of injuries [3].

KFS was first described by Andre Feil and Maurice Klippel in 1912; the exact cause and pathogenesis of it are unclear, but it is thought to be caused due to a genetic mutation in the TGF-B (transforming growth factor beta) family of proteins. The most likely associated genes are GDF (growth differentiation factor) 6 and GDF 3/mesenchyme homeobox 1 [4], and the exact mechanism is still unclear.

KFS is thought to be caused embryologically by the failure in normal vertebrae segmentation during the fourth week of gestation [5]. It is associated with other somite defect anomalies in the urogenital system, cardiovascular system, and skeletal system [6]. Multiple associated presentation is possible, but in our case, there was an isolated spinal deformity alone. During the management, a 2D echo was done to rule out congenital cardiovascular anomalies, and an ultrasound of the pelvis was done to rule out anomalies in the urogenital system. Hearing assessment ruled out possibilities of deafness, and otolaryngologist opinion was sought to rule out any sort of low-frequency pure tone loss.

The prevalence of KFS is stated to be one in 42,000 live births with a female preponderance of 60%, the fusion most commonly occurring at the cervical level C2-C3 as quoted in most major studies, with multilevel fusion being highly uncommon accounting for less than 10% of all cases with KFS [6].

We described a case with a multilevel fusion of four cervical vertebrae (C3-C7) with a single fused spine. Owing to the altered biomechanics of the spine secondary to the fusion of the vertebrae, the patient developed severe cervical myelopathy leading to quadriplegia following trivial trauma.

Patients with KFS are radiologically divided into three categories: types I-III. Type I was defined as those having a single fused cervical vertebral segment; type II was defined as those having multiple noncontiguous, congenitally fused segments; and type III was defined as those having multiple contiguous, congenitally fused cervical segments [7]. The patient reported in this study falls under type III of KFS.

**Figure 3:**
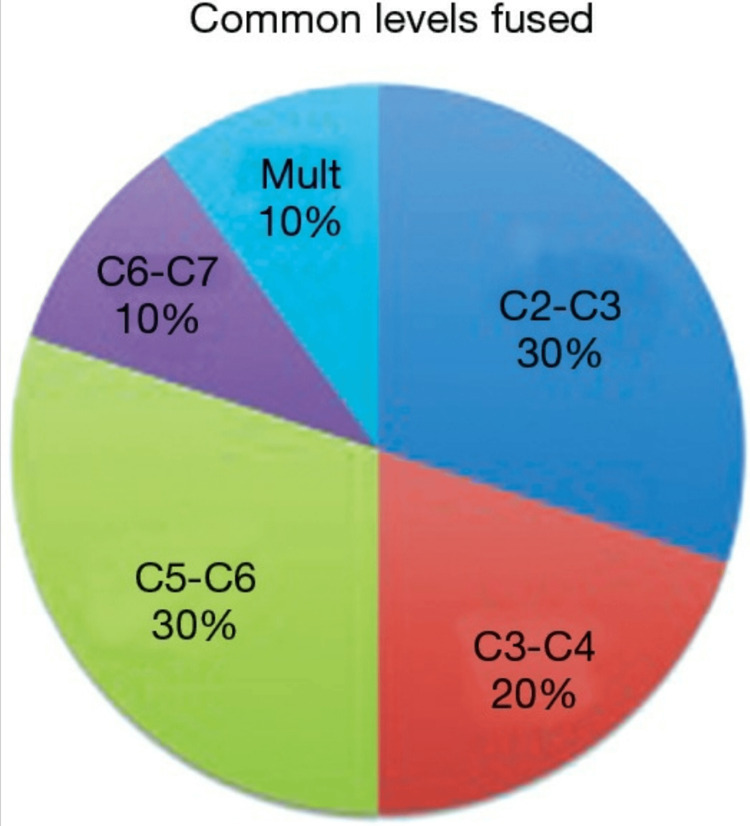
Pie chart showing the prevalence of Klippel-Feil syndrome with the fusion of different levels of cervical vertebra (C)

In patients with KFS, the prevalence of cervical vertebral fusion (Figure 3) is as follows: 30% seen at C2-3, 20% at C3-4, 30% at C5-6, 10% at C6-7, and 10% at multiple cervical vertebrae. Our case had a rarity of fusion of more than four vertebrae [7].

Diagnosis in KFS is established radiologically, with preliminary evaluation by x-ray neck followed by CT and MRI. As our case had an active spinal cord injury suspicion, we went forward with an MRI (Figure 1). Other malformations of Arnold-Chiari and diastematomyelia were ruled out [8].

KFS is usually diagnosed at birth by the phenotypic features. The management involves a multidisciplinary care team with a pediatrician. In a few instances, it goes undiagnosed till adulthood in cases with isolated spinal deformity, similar to our case. In those cases, the approach to management is basically on the clinical assessment of acute presentation. Patients with persistent neurological pain, myelopathy, new-onset muscle group weakness, and documented spinal instability are operative candidates. Our patient had a new-onset weakness with no active instability, and compression was not very significant for active surgical intervention. Hence, we took the nonoperative management of observation. He improved and was able to be discharged with home care. If required, the option for surgical management includes anterior cervical fusion or corpectomy with the placement of either synthetic or bone graft, but this was not required in our case [9].

We advised this case with activity restriction to avoid spinal cord injury. On reviewing the literature, it was mentioned that spinal cord fusion below C3 requires no activity restriction and can engage in contact sports. But in our case, though the fusion level was below the C3 at multiple levels and a trivial trauma resulted in spinal cord injury, this is against the literature, which is the rarity of the report. Based on this, we advised activity restriction for cases of KFS [9].

Similar cases of spinal cord injury with KFS have been reported, but none had a traumatic fall as in our case. Both of them had been treated surgically with anterior fusion. In our case, we went with conservative management with good results based on the clinical assessment and involvement of spinal injury [10].

## Conclusions

The authors would like to conclude that screening and identification of KFS in young children is essential as counseling for lesser strenuous activity at work in the future can avoid any complications and eventually result in better neurological outcomes.
